# Body Design or Behavior? What Explains the Performance of Slender-Billed Gulls (*Chroicocephalus genei*) Feeding on Brine Shrimp (*Artemia* sp.) in Salt Pans?

**DOI:** 10.3390/biology14101331

**Published:** 2025-09-26

**Authors:** Maud de Saint Seine, Lyse Hannier, Vincent Bels, Nicolas Schtickzelle, Michel Baguette

**Affiliations:** 1 Earth and Life Institute, UCLouvain, 1348 Louvain-la-Neuve, Belgium; maudstseine@gmail.com (M.d.S.S.); lyse.hannier@gmail.com (L.H.); nicolas.schtickzelle@uclouvain.be (N.S.); 2 Muséum National d’Histoire Naturelle, Institut de Systématique, Évolution, Biodiversité, UMR 7205 Muséum National d’Histoire Naturelle, CNRS, Sorbonne Université, EPHE, Université des Antilles, F-75005 Paris, France; vincent.bels@mnhn.fr; 3 CNRS, Station d’Écologie Théorique et Expérimentale, UAR 2029, F-09200 Moulis, France

**Keywords:** form–function, performance, functional morphology, food acquisition behavior, food metabolic rate, behavior convergent evolution, behavior phylogenetic conservation

## Abstract

(1) Background: Understanding the evolution of the form–function relationship requires identifying the selection pressures acting on individuals. The paradigm of Arnold provides a useful framework to infer how the natural selection acting on phenotypic traits can modulate individual fitness. Despite the theoretical advance, experimental studies of individual performances that explicitly address form, i.e., the phenotypic integration of functional morphology (body design and mechanics) and of behavior, are still rare. (2) Methods: Slender-billed gull food acquisition behaviors were video recorded in the salt pans of Salin de Giraud, Camargue, where brine shrimp are their main prey. We averaged the food intake rate over 21 individuals. We computed the mean hourly energy intake of an average gull by multiplying the mean hourly prey intake rate by the weight and energy content of a brine shrimp. We used this mean hourly energy intake to investigate the time needed by an average slender-billed gull individual to acquire the energy required to achieve their daily field metabolic rate. We computed the food metabolic rate of slender-billed gulls by using the model of Dunn et al. In addition, using slow-motion video sequences, we perform a functional and integrative analysis of three performances associated with food acquisition behaviors, i.e., locomotion, food capture, and food transport. (3) Results: We demonstrate that the energy assimilated by this performance is sufficient to cover less than 6 h of an adult gull’s metabolic food rate during its breeding season. We show that brine shrimp capture by gulls does not involve the use of specialized morphological structures but rather involves a particular behavioral sequence that invariably associates a mode of locomotion, a mode of capture and a mode of transport of the prey from the beak to the pharynx. The comparison of this sequence to the register of food acquisition behaviors used by other Charadriiformes reveals its similarity with behaviors that are used by two shorebird species (*Phalaropus fulicarius* and *P. lobatus*) also feeding on prey captured from saltwater surfaces. (4) Conclusions: Altogether, our study supports (1) a causal chain in which performance results from the interaction between morphological structures and behaviors and (2) the idea that the performance peak of a realized phenotype can be reached by using the best combination of behaviors, either by convergent evolution or by their conservation among those available in a phylogenetically determined register.

## Simple Summary

How animals perform vital tasks for their survival and reproduction is a crucial question to understand their current lifestyle and its evolution. Here we focus on the food acquisition by adult gulls feeding on small shrimp in salt pans in Camargue, France. The rate at which these gulls capture and ingest shrimp is extremely high. We calculate that this rate is high enough that it covers the energy requirement of an adult gull at its peak during the breeding season in less than 6 h. By analyzing slow-motion videos of feeding gulls, we were able to show that this performance is not achieved using particular, specialized morphological structures but rather by optimizing the suite of behaviors associated with locomotion, food capture, and food transport. This suite of behaviors is comparable to that used by two species of phalaropes that are not gulls but shorebirds, which also feed on prey captured at the saltwater surface. This raises the question of the similarity of these food acquisition behaviors in species belonging to different lineages, either due to their convergent evolution in the face of common environmental constraints or due to their maintenance during the diversification of lineages along the tree of life.

## 1. Introduction

Macroevolutionary analyses aiming at exploring form–function relationships investigate how phenotypes are organized to perform relevant ecological functions by focusing on species-specific, broad ecological characteristics like, i.e., trophic levels, dietary resource types, and foraging behaviors (e.g., [1,2]). However, the understanding of these form–function relationship evolutions requires identifying what the selection pressures acting on individuals are, and it is therefore necessary to downscale these broad ecological characteristics into their constituent phenotypic traits. The paradigm of Arnold [3,4] provides a useful framework to infer how the natural selection acting on phenotypic traits can modulate individual fitness. According to this paradigm, pertinent phenotypic traits (e.g., morphological, physiological, and behavioral) are shaped by natural selection to optimize individual performance [3,4], i.e., any quantitative measure of how well an organism performs an ecologically relevant task that is vital for its fitness [5]. The ecological functions fulfilled by the individual will therefore result from two processes: the integration of its phenotypic traits and the optimization of its performance, with evolutionary feedbacks between them. Despite the availability of this theoretical framework, experimental studies of performances that explicitly address form, i.e., the phenotypic integration of functional morphology (body design and mechanics) and of behavior, are still rare [6], probably because separating the “why” question (behaviors) from the “how” question (the design and mechanics of the body), and vice versa, is very complex, as these two questions are so closely intertwined [6,7,8,9]. We define behavior here as the internally coordinated responses (actions or inactions) of whole living organisms to internal and/or external stimuli, excluding responses more easily understood as developmental changes [10]. We consider behavior as a phenotypic trait at the same level as morphology or physiology, according to Emerson and Arnold [4] and Green et al. [6], and unlike Bels et al. [8] and Garland and Losos [11], who use behavior as a way to capture how an organism’s performance (usually measured as a maximum value in the lab) is realized in natural conditions (similar to the “realized ecological niche” [12]) [6].

Predator-prey interactions might be excellent model systems in studies of behavior and functional morphology because both predators and prey can evolve extreme adaptations due to the coevolutionary “arms race” between them [13]. The extreme specialization for capturing prey or escaping predators could then be used to test the relative roles of functional morphology and behavior in driving performances in nature. Here we use a couple of predator-prey species under completely natural situations to investigate how the body design of a predator and its behavioral register are integrated to shape its performance in prey acquisition. Gulls, i.e., bird species of the family Laridae, are characterized by the uniformity of their body plan organization [14]. In this family of about 50 species [14], the proportions of appendage size relative to body size remain fairly constant, with the exception of rather slight variations in length and thickness of the beak. This constancy in morphology contrasts with what happens in some other families belonging to the same order of Charadriiforme as the Laridae, and particularly in the shorebirds (Scolopacidae and Charadriidae), in which the size and shape of the legs, toes, wings, and especially the beak show huge interspecific variation. Despite this homogeneity of morphology, gull species generally show great inter- and intraspecific versatility in their diet (e.g., [14,15,16,17,18,19]). This translates, for example, into a very high adaptability to the use of foods of human origin, including the use of specialized behaviors such as kleptoparasitism to steal meals from the hands of inattentive tourists in their seaside holiday resorts [20]. However, certain species of Laridae exhibit a strong dietary specialization.

This is the case of the slender-billed gull (*Chroicocephalus genei*) that feeds mainly on small invertebrates and fish [21,22], and especially on brine shrimp (*Artemia* sp.) on salt pans during its breeding season [23,24]. We have here an exceptional, quasi-experimental situation to study if and how the effectiveness of a performance depends rather on body design or behavior. Indeed, brine shrimp are the only predictable prey on which gulls can feed in this very particular environment. Adult brine shrimp measure a maximum of 10 mm long and weigh a maximum of 10 mg [25]. These ostracods are extremely abundant in salt pans, reaching, for instance, at adult summer peak density, a biomass of 1600 kg/ha in the Mediterranean salinas of Salin de Giraud, France [23].

Although there have been qualitative field observations of slender-billed gulls feeding on brine shrimp, it is unclear how this diet is sufficient to satisfy all their metabolic requirements. We will first evaluate to what extent the performance of gulls feeding on brine shrimp, as measured by their food intake rate, allows them to cope with their daily energy requirements. For this, we will use the food metabolic rate (FMR), which is the total sum of energy that a free-ranging animal metabolizes over a specified time [26]. By using the model estimating field metabolic rates (FMR) in seabirds of Dunn et al. [26], we will demonstrate that the energy assimilated by this performance, i.e., the food intake rate, is more than sufficient to cover an adult gull FMR during three different periods of its breeding season. We will then look at how slender-billed gulls acquire this very particular prey and show that brine shrimp capture does not involve the use of specialized morphological structures but rather involves a particular behavioral sequence that associates a mode of locomotion, a mode of capture, and a mode of transport of the prey from the beak to the pharynx. The comparison of this sequence to the register of food acquisition behaviors used by other Charadriiformes (shorebirds: Baguette et al. [27]) reveals its convergence with a similar sequence of locomotion, capture, and food transport behaviors that is used by a shorebird species also feeding on prey captured on saltwater surfaces. Altogether, our study supports (1) a causal chain in which a performance results from the interaction between morphological structures and behaviors according to Emerson & Arnold [4] and Green et al. [6], and (2) the idea that the performance peak of a realized phenotype [9] can be reached by using the best combination of behaviors either by convergence evolution or by their conservation among those available in a phylogenetically determined register.

## 2. Materials and Methods

### 2.1. Data Collection

Slender-billed gull food acquisition behaviors were video recorded during a one-week field session between 1 April and 8 April 2024, in the salt pans of Salin de Giraud, Camargue, France. We worked at the They de St. Ursule lagoon (43.359290° N, 4.78394° E, see map in Supplementary Material), in which the salinity of water is between 140 and 270 g/L [24]. Data were collected as follows: observers avoiding bird disturbance were positioned at distances ranging from 5 m to 20 m, in or behind a car, and filmed using an Olympus OMD-EM1x (Olympus Corporation, Tokyo, Japan) with an M.Zuiko Digital ED 150–400 mm F4,5 lens (Olympus Corporation, Tokyo, Japan), which provides magnification up to 40 times. Focal individuals [28] were selected (among flocks when necessary) and filmed at a speed of 60 fps. Care was taken to avoid filming the same individual twice, which guarantees the independence of the data on food intakes between individuals. We ended with 11 video sequences that contain usable prey capture data by 21 focal individuals. Besides these video recordings, we observed and photographed slender-billed gulls feeding on brine shrimp at the same place in April 2021, September 2021, and July 2023, which allows us to generalize the conclusions of video recordings during the period of presence of these migratory birds on their breeding sites in Camargue.

### 2.2. Data Analysis

#### 2.2.1. Intake Rate

In our study site and at this period of the year, adults and nauplii of brine shrimp are the main available invertebrate prey for foraging birds [23], and the only ones to be near the surface. Due to their positive phototaxis (e.g., [29]), brine shrimp move close to the water surface, where they are pecked by swimming gulls with the tip of their beak without having to immerse their head. The movement of the gull associated with capture is clearly identifiable: while swimming, the bird tilts its head, quickly pushes the tip of its beak into the water, and then pulls it out by raising its head. Beak movements associated with the transport of the prey towards the pharynx are clearly visible, as well as prey swallowing. Accordingly, we viewed each sequence for our 21 individuals and counted the number of events starting with an immersion of the tip of the beak and ending with swallowing movements. Each of these events is considered a capture. We summed the number of these captures over the entire video sequence for a given individual. By dividing the number of captures by the duration of the video sequence, we inferred the individual’s intake rate.

#### 2.2.2. Field Metabolic Rate

To investigate the field metabolic rate of slender-billed gulls feeding on brine shrimp at the They de St. Ursule lagoon, we averaged the intake rate over our 21 individuals. Then we computed the mean hourly energy intake of a gull by multiplying the mean hourly prey intake rate by the weight and energy content of a brine shrimp (Equation (1)).

(1) HourlyEnergyIntake=HourlyPreyIntakeRate×PreyMeanWeight×PreyMeanEnergyContent

Brine shrimp adult maximum wet weight is 10 mg [25], with a water content of 87% [30], giving a dry weight of 1.3 mg. Energy content is 22 kJ/g of dry weight [30]. We used this mean hourly energy intake to investigate the time needed by slender-billed gull individuals to acquire the energy required to achieve their daily field metabolic rate (FMR). We computed the FMR of slender-billed gulls at the They de St Ursule lagoon by using the model of Dunn et al. [26], which provides FMR for any seabird populations. We used the ‘Seabird FMR calculator’ [26], which is a dedicated app located at https://ruthedunn.shinyapps.io/seabird_fmr_calculator/ (accessed on 1 July 2024). We input into the model the mean mass of slender-billed gulls (285 g, [22]) and the latitude of the lagoon (43°).

### 2.3. Behavioral Data

We used the approach we designed for shorebirds [27] to perform a functional and integrative analysis of behaviors associated with food acquisition. Food acquisition is divided into three successive stages: foraging, feeding, and swallowing. The foraging stage concerns the different behaviors associated with food detection and capture, i.e., locomotion behaviors (how do birds move in suitable foraging habitats) and capture behaviors (how do birds locate and catch the prey). The feeding stage encompasses the behaviors used to handle the prey and to transport the prey into the pharynx. In the swallowing stage, the prey enters the digestive tract. We viewed the video sequences of our 21 focal individuals and detailed the behaviors associated with these three stages of food acquisition. We compared them to our behavioral register established on another group of Charadriiformes, the shorebirds.

## 3. Results

### 3.1. Hourly Energy Intake Rate

The number of captures, the duration of the observation, and the resulting intake rate (captures/min) for the 21 focal individuals are shown inn Table 1. The mean intake rate is 58.2 ± 17.7 captures/min (min. 27.3, max. 921.9), which means a hourly prey intake rate of 3492 brine shrimp/hour. This food intake rate corresponds to our observations made in other years and at other times of the year.

We solved Equation (1):

3492 × 0.0013 × 22 = 99.87 kJ/h, which provided us with an estimate of the hourly energy intake rate by slender-billed gull feeding on brine shrimp in the salt pan of Salin de Giraud.

### 3.2. Catching Time Needed to Cover the Energetic Requirement of FMR

We used first the model of Dunn et al. [1] to estimate the FMR of slender-billed gulls at three different phases of their breeding period (Table 2). Classically, the FMR grows according to the development of the chicks; it is minimum during the incubation phase, it increases when the pulli (young birds that cannot yet fly) are fed in the nest, and it is maximum when juveniles are in the crèche (Table 2).

Next, we computed the time that an individual gull would need catching brine shrimp to cover these three different FMRs (Table 2). The increase in food demand by chicks over the reproduction period results in a 74% increase in the daily time a gull would spend fishing brine shrimp.

### 3.3. Food Acquisition Behaviors

**Figure 1:**
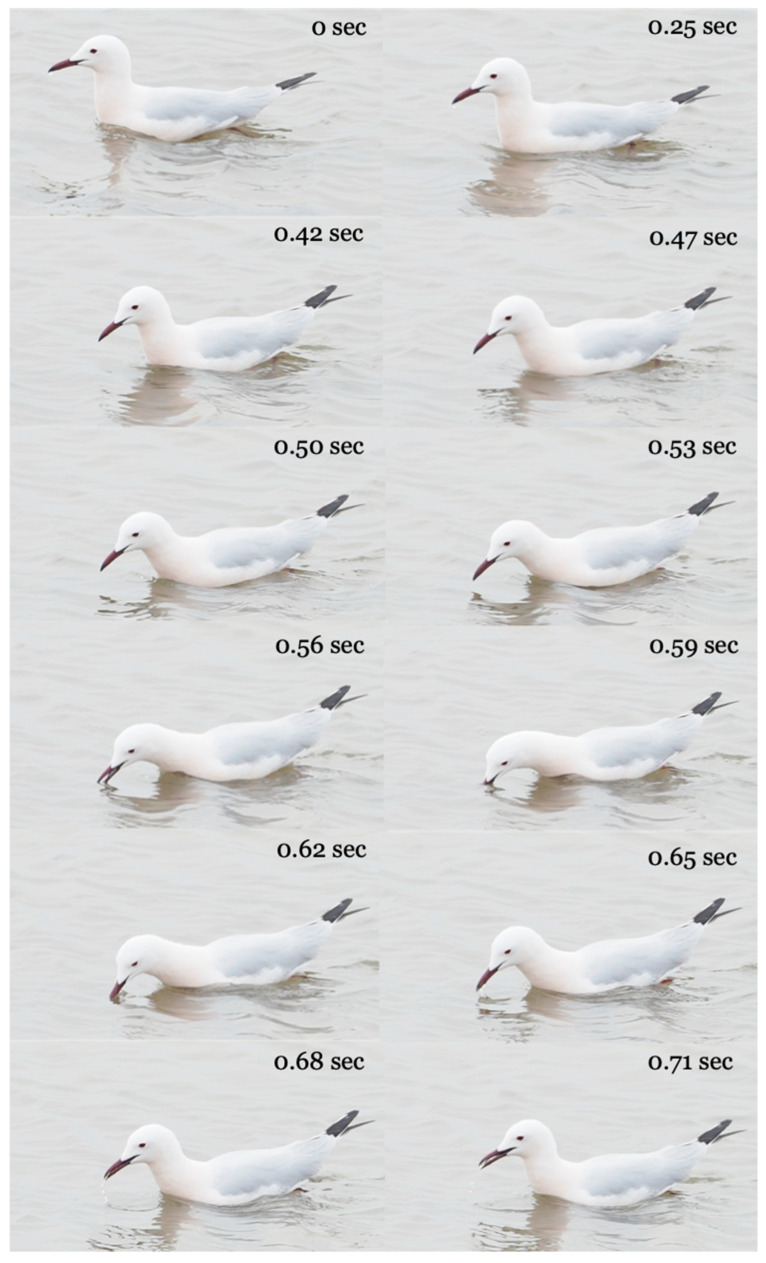

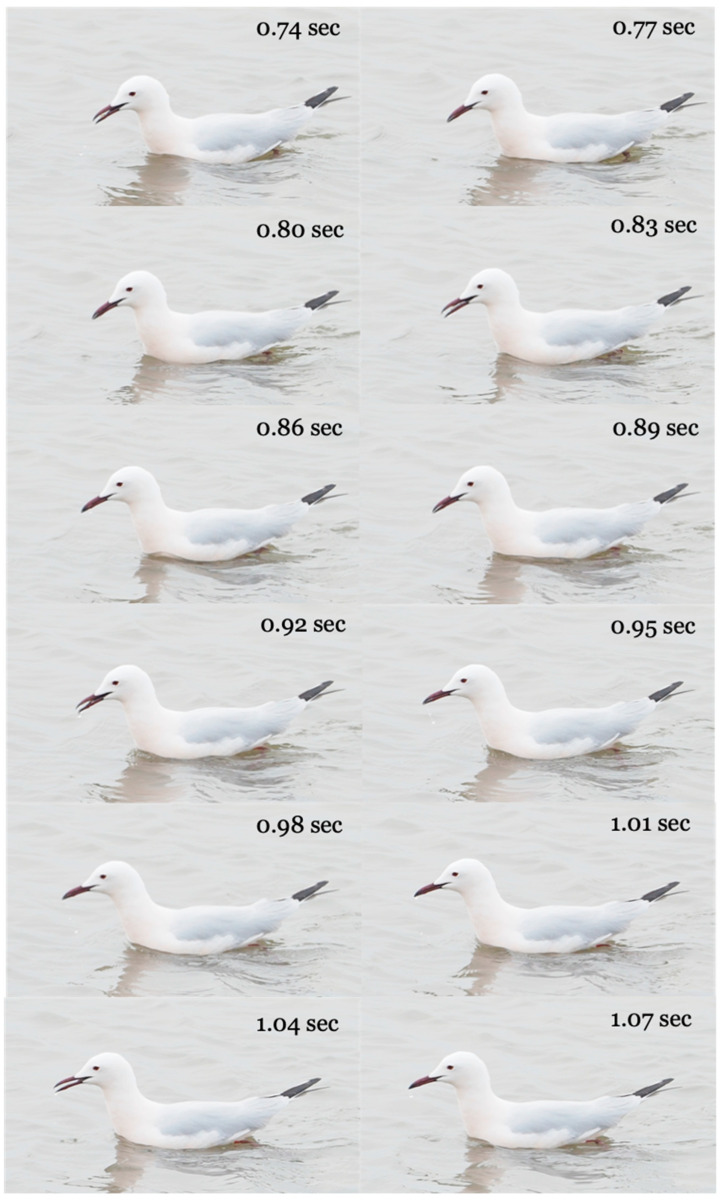
Illustration of the food acquisition behavior of a slender-billed gull feeding on a brine shrimp. The capture behavior begins at 0.42 sec and ends at 0.62 sec. Transport of the captured shrimp from the tip of the beak towards the pharynx begins at 0.65 sec. The transport of the shrimp ends at 0.95 sec, with the spitting of water droplets used for transport.

Locomotion behavior associated with the foraging stage is continuously swimming, according to the repertoire of Baguette et al. [27]. Gulls move continuously by vigorously moving their legs in the water and change direction frequently and abruptly (Figure 1). As such, changes in direction are most often followed by the capture of a shrimp, we assume that they are triggered by prey visual contact. Capture behavior is pecking: the gull in motion picks the shrimp without stopping its continuous swimming. (Figure 1). The prey handling behavior that initiates the feeding stage is absent: upon capture, transport begins to bring the shrimp from the tip of the beak to the pharynx (Figure 1).

**Figure 2:**
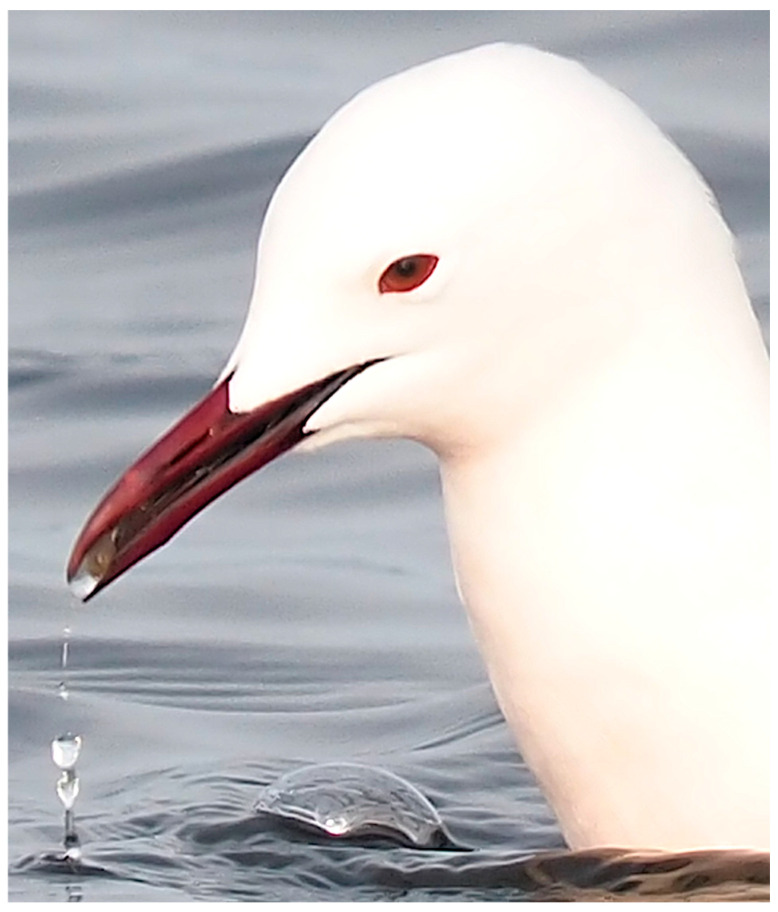
Close up of the head of a slender-billed gull (*Chroicocephalus genei*) after capture of a brine shrimp (*Artemia* sp.). The shrimp is clearly visible within a droplet of water between the upper and lower mandibles. Picture by Michel Baguette.

Transport behavior is clearly achieved by surface tension: close-up images indicate the presence of prey in a water droplet that is inserted between the upper and lower mandibles (Figure 2).

**Figure 3:**
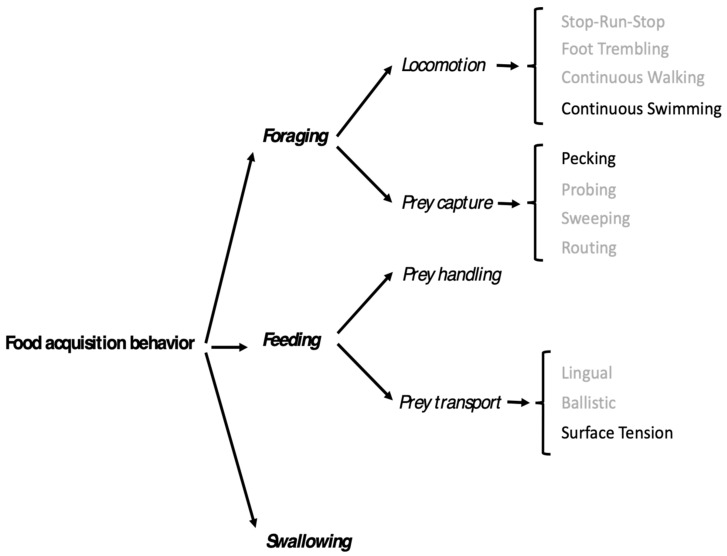
Traitgram of food acquisition behaviors used by slender-billed gulls feeding on brine shrimp (in black) following the hierarchical classification of food acquisition behaviors [27]. Grey color indicates possible behaviors as described in [27], black color the behaviors used by slender-billed gulls.

In addition, the swallowing movements associated with the swallowing stage are accompanied by the release of drops of water from the tip of the beak. The food acquisition behavior of slender-billed gulls is summarized in Figure 3.

## 4. Discussion

Here we investigate how the body design and the food acquisition behaviors of a predator (the slender-billed gull) interact to optimize its food intake rate on its prey (brine shrimp) and to what extent this performance is sufficient to cover its daily energetic requirements. In a first step, we quantified the energetic requirement of gulls during their breeding period and assessed whether the daily brine shrimp intake rate could cover these energy costs. Our calculation showed that the prey-catching time needed by an individual to cope with the energetic demand of the different phases of the breeding period seems rather coherent and, thus, that a diet composed of brine shrimp only could be enough to meet these needs. However, here we must mention that it is very difficult to obtain reliable data on the physiology of brine shrimp living in natural conditions [31]. There are so many contradictory studies on laboratory strains that we have favored parameters measured on wild individuals [25].

Notwithstanding these difficulties in parameter estimate, our measurement of the daily energy acquisition of the gulls observed at our study site shows that it is sufficient to cover the energy requirements required during the breeding period, as predicted by the FMR modeling approach of Dunn et al. [26]. Even if the feeding period of the young requires a significant increase in catching time by the parents, their rate of intake of brine shrimp allows them a sufficient energy supply to meet these needs. Brine shrimp show a huge seasonal variation in the salt pans of Salin de Giraud: they are only present in the form of dormant eggs (cysts) on the bottom of salinas during winter, while adults reach a density of 16,000 individuals/m^2^ in open water at their summer peak density [23]. Added to this enormous density is positive phototaxis, which causes brine shrimp to be attracted to the surface to feed on photosynthetic algae and cyanobacteria (e.g., [29]), and a lack of adaptation to resist predation [23]. Even if this energy-rich prey is extremely abundant and easy to capture, the fact remains that to cover their FMR during the breeding season, slender-billed gulls must capture them at a sustained rate. Our measurements provided impressive food intake rates, with a mean of 58 captures/min, peaking at 92 captures/min.

We mention here that we use in our calculation the maximum weight of adult brine shrimp available in the literature, which is the one that will yield the maximum energy return to the feeding gull. It is obviously impossible to measure the size of each shrimp caught by a feeding gull. In addition, the size distribution within shrimp populations is expected to be highly variable because the brine shrimp is a multivoltine species with several overlapping generations in a year [23], which means that both nauplii and adults of different sizes would be simultaneously available for predation. However, we wish to highlight two points to support our conclusions: (1) we believe that slender-billed gulls will maximize their food intake rate by preferentially hunting large shrimp, and (2) even if the shrimp were half the size used in our calculation, the hunting periods necessary for a bird to cover its FMR remain entirely achievable over the course of a day.

Our energetic and behavioral analysis is based on a relatively limited set of video sequences (21 individuals). Efforts were made to avoid re-sampling: we tried to film individuals only once. Because birds were not marked, we filmed no more birds, which would have prevented us from being sure they were different individuals within a one-day session. Given the high number of slender-billed gulls feeding on the They de Saint Ursule during our field session in April 2021 (around a hundred individuals), coupled with the fact that we filmed in different areas of the lagoon from one day to the next, it is unlikely that we recorded the same individual twice. Furthermore, visual observations and photographs confirm the results of the video analyses, both during the April 2021 session and during the other sessions carried out at the same location in September 2021 and July 2023. We can also mention that the behaviors observed in our study lagoon are similar to those practiced by slender-billed gulls on other salt pans in Salin de Giraud. Moreover, individuals modify their foraging trajectories or interrupt their capture behaviors depending on the arrival or departure of their conspecifics, or even their spatial proximity. These behaviors are further modified depending on the presence of predators. We only have unquantified observations of these behavioral modifications depending on the broader social context, but it would be very interesting to integrate them into future studies.

We can now turn to the central question of our paper, i.e., the interaction between the body design and the food acquisition behaviors in allowing the gull to achieve this performance. Beginning with morphology, we might expect that slender-billed gulls have a more slender beak than other gulls. However, these gulls are not comparable with other gulls with a white head; slender-billed gulls are now considered as members of the *Chroicocephalus* genus, most members of which are masked gulls [14]. The comparison of beak length-depth ratios between the slender-billed gull and other *Chroicocephalus* species (Table 3) shows that the slender-billed gull does not fully deserve its vernacular name: even if it has the slenderest beak among its congeneric species, it is not by much. It would be very interesting to further this analysis of beak “slenderness” by collecting data on a sufficient number of individuals in order to use relevant statistical models to quantitatively test the level of interspecific differences. This could shed light on the level of difference that would be necessary to support (or reject) the hypothesis of morphological adaptation.

The diet composition of other members of the *Chroicocephalus* genus is rather generalist (Table 3), and the slender-billed gull seems rather special by the fact that it does not feed much on offal. It shares this particularity with Bonaparte’s gull (*Chroicocephalus philadelphia)* and the gray-headed gull (*Chroicocephalus cirrocephalus*).

The stages of the slender-billed gull’s food acquisition behavior when feeding on brine shrimp are quite distinct and do not show variation. As the density of shrimp is very high, the sequence of gull locomotion and prey capture, handling, and transport occurs very quickly. Visual detection of prey is evident in the sudden and rapid changes in direction of the swimming bird. This fluidity in the sequence of these successive stages undoubtedly explains the very high food intake rate we report here. Overall, the food acquisition behavior of the slender-billed gull is completely similar to that which we described in shorebirds, the grey phalarope (*Phalaropus fulicarius*) and the red-necked phalarope (*P. lobatus*) feeding on floating invertebrates: locomotion by continuous swimming, capture by pecking on or near the sea surface, without immersion of the head, and transport of the prey from the tip of the beak to the pharynx by surface tension [27]. It is salient to note that this similarity in food acquisition behavior with phalaropes has also been explicitly noted in another member of the genus *Chroicocephalus*, the Bonaparte’s gull (*C. philadelphia*) [33]. The very high food intake rate achieved by slender-billed gulls does not seem to be due to the evolution of a specialized morphological structure, as its beak length-depth ratio is quite comparable to that of its congeneric species. Rather, it seems to be due to the optimal use of an existing structure by highly effective behavioral sequences in this environmental context of high density of such small prey that they require a very quick acquisition. Putting this result in the context of the causal chain that arises from the paradigm of Arnold [3,4] indicates that a performance—in this case the food intake rate, which is quantifiable and has a direct effect on the fitness of the individual—is achieved by the interaction between a series of stereotyped behaviors and the morphology of the beak of the bird. We also show that the integration of these two phenotypic traits (the morphology and mechanics of the beak on the one hand and the behavioral sequences on the other hand) optimizes the performance of food intake rate so as to be able to cover the FMR of the gull even at its peak during the maximum demand of offspring.

## 5. Conclusions

Altogether, our study supports a causal chain in which a performance results from the interaction between morphological structures and behaviors. Here the performance of slender-billed gulls feeding on brine shrimp is not achieved using particular, specialized morphological structures but rather by optimizing the suite of behaviors associated with locomotion, food capture, and food transport. The similarity of this suite of behaviors with those used by species from a distinct lineage (*Phalaropus* spp.) in the same environmental context supports the idea that the performance peak of a realized phenotype can be reached by using the best combination of behaviors, either by convergent evolution or by their conservation among those available in a phylogenetically determined register.

## Figures and Tables

**Table 1:** Number of captures, duration (s) and intake rate (capture/min) for the 21 focal individuals of slender-billed gull feeding on brine shrimp at the They de St Ursule lagoon.

Individual	Sequence Reference	Duration (s)	Captures	Intake Rate (Capture/min)
1	40071003	61	42	41.3
2	40071003	15	20	80
3	40071005	32	49	91.9
4	40071008	33	26	47.3
5	40071009	31	33	63.9
6	40071010	33	19	34.5
7	40071011	39	36	55.4
8	40071066	28	27	57.9
9	40071066	11	5	27.3
10	40071066	26	16	36.9
11	40071066	4	5	75
12	40071066	23	31	80.9
13	40071020	11	9	49.1
14	40080005	15	11	44
15	40080005	27	24	53.3
16	40080005	10	9	54
17	40080009	63	76	72.4
18	40080096	7	7	60
19	40080096	8	6	45
20	40080096	8	9	67.5
21	40080096	5	7	84

**Table 2:** Field metabolic rate (kJ/Day) at three phases of the reproduction period and catching time needed by a gull feeding on brine shrimp to cover these energetic requirements.

Reproduction Phase	Field Metabolic Rate (kJ/Day)	Daily Time of Brine Shrimp Catching (h)
Incubation	311.44	3.07
Brood	490.70	4.54
Crèche	556.01	5.34

**Table 3:** Beak length and depth (mm), beak length-depth ratios, and diet composition of representatives of the genus *Chroicocephalus*. Higher beak length-depth ratios are indicative of proportionally longer and slimmer beaks. Data source: Avonet, Tobias et al. [32] for measurements, Burger et al. [14] for diet composition.

Species	Beak Length (mm)	Beak Depth (mm)	Length-Depth Ratio	Diet Composition
*C. brunnicephalus*	47.4	11.1	4.3	Fish, shrimp, insects, offal
*C. bulleri*	41.5	7.9	5.2	Fish, earthworms, insects, aquatic invertebrates, offal
*C. cirrocephalus*	47.5	10.2	4.6	Fish, invertebrates, eggs, kleptoparasitic, scavenger
*C. genei*	52.1	9.4	5.5	Fish, aquatic invertebrates including *Artemia*
*C. hartlaubii*	39.5	8.9	4.4	Generalist
*C. maculipennis*	41.4	8.9	4.6	Generalist
*C. novaehollandiae*	39.6	10.2	3.9	Generalist
*C. philadelphia*	35.0	6.6	5.3	Fish, insects
*C. ridibundus*	43.4	7.9	5.5	Generalist
*C. serranus*	41.9	9.7	4.3	Generalist

## Data Availability

The raw data supporting the conclusions of this article will be made available by the authors, without undue reservation.

## Supplementary Materials

The following supporting information can be downloaded at: https://www.mdpi.com/article/10.3390/biology14101331/s1, Figure S1: Map of the They de Saint Ursule study site.
