# Supplementary figures and images for: Body Design or Behavior? What Explains the Performance of Slender-Billed Gulls (Chroicocephalus genei) Feeding on Brine Shrimp (Artemia sp.) in Salt Pans?
Source: Biology (Basel). 2025 Sep 26;14(10):1331. doi: 10.3390/biology14101331 (PMC12561011; doi:10.3390/biology14101331)

Supplementary Material Figure S1: Map of the They de Saint Ursule study site.

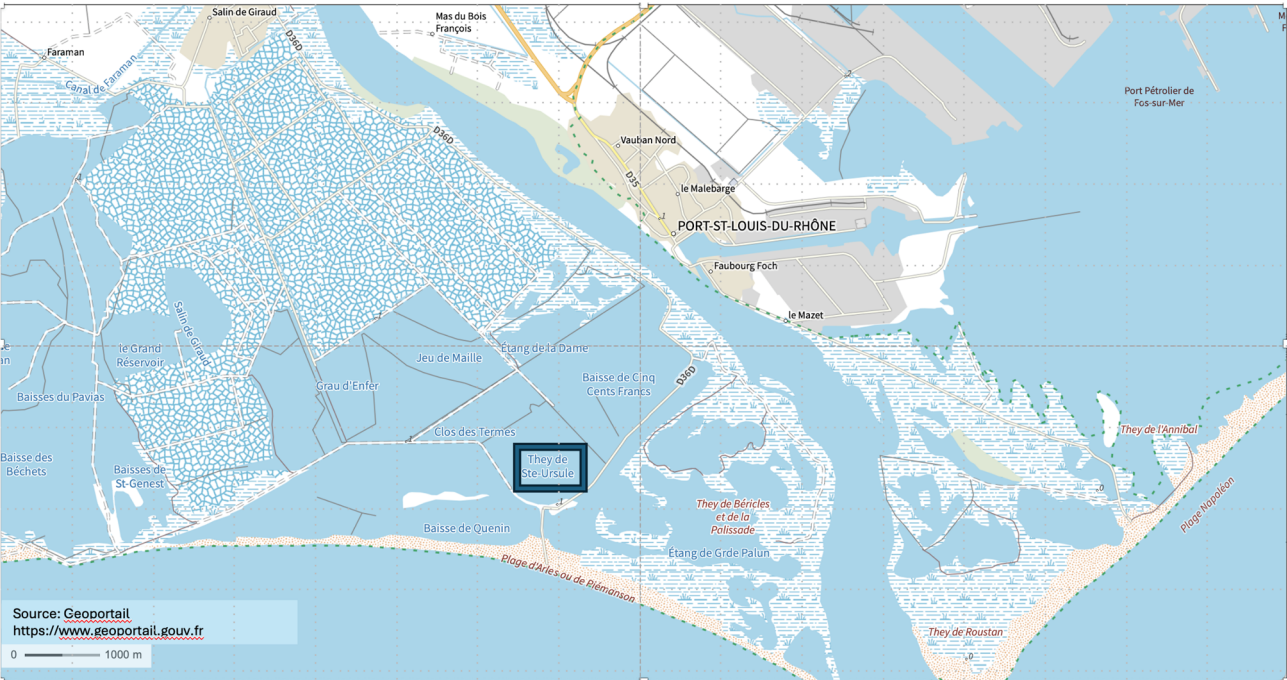

Supplement: Supplementary file 1 [file biology-14-01331-s001.zip › biology-3780188-supplementary.pdf]
